# Supplementary material for: High prevalence and co-infection of high-risk Human Papillomavirus genotypes among unvaccinated young women from Paraguay
Source: PLoS One. 2023 Apr 6;18(4):e0283542. doi: 10.1371/journal.pone.0283542 (PMC10079089; doi:10.1371/journal.pone.0283542)
Supplement: Attachment — Submitted filename: Response to Reviewers 23th Dec PONE-D-22-28769.docx [file pone.0283542.s001.docx]

December 23^th^, 2022

**Manuscript number:** PONE-D-22-28769

**Manuscript title:** "HIGH PREVALENCE AND CO-INFECTION OF HIGH-RISK HUMAN PAPILLOMAVIRUS GENOTYPES AMONG UNVACCINATED YOUNG WOMEN FROM PARAGUAY”

Dear Academic Editor Andrea Giannini

Please find attached our revised manuscript for PLOS ONE by Bobadilla ML et al.

We are grateful to you and the reviewers for the very helpful comments and the opportunity to resubmit, and per your instructions, the new version contains new text that address concerns noted in the reviews.

In addition to the revised manuscript, the Response to Reviewers document which contains a detailed point-by-point reply to the requests and comments from the reviewers is also attached.

We look forward to your response and sincerely hope I hope you and the reviewers will be satisfied with the revised manuscript and will find it suitable for publication.

This study was supported by the National Council of Science and Technology (CONACYT) through the PROCIENCIA Program with resources from the Fund for Excellence in Education and Research (FEEI) (RESERCH 15-INV-200), Research, Education and Biotechnologies Applied to Health (IEBAS) Project – MERCOSUR Structural Convergence Fund (FOCEM), COF 03-11, Coordination for the Improvement of Higher Education Personnel (Coordenação de Aperfeiçoamento de Pessoal de Nível Superior - CAPES) - Finance Code 001, Fundação Carlos Chagas Filho de Amparo à Pesquisa do Estado do Rio de Janeiro (FAPERJ), Oswaldo Cruz Institute and Conselho Nacional de Desenvolvimento Científico e Tecnológico (CNPq).

Sincerely yours,

María Liz Bobadilla

Lab. of Immunology, Central Laboratory of Public Health/Minister of Public Health and Social Welfare, Asunción, Paraguay.

Lab. De Virologia Molecular IOC/FIOCRUZ, Rio de Janeiro, Brazil.

**JOURNAL REQUIREMENTS:**

1. Please ensure that your manuscript meets PLOS ONE's style requirements, including those for file naming.

We already have done.

2. Thank you for submitting the above manuscript to PLOS ONE. During our internal evaluation of the manuscript, we found significant text overlap between your submission and previous work in the Introduction section.

The significant text overlap with previous work in the introduction were taken from the available literature at PUBMED. We added in the manuscript the authors and cited the corresponded reference.

3. Please include a complete copy of PLOS’ questionnaire on inclusivity in global research in your revised manuscript. Our policy for research in this area aims to improve transparency in the reporting of research performed outside of researchers’ own country or community. The policy applies to researchers who have travelled to a different country to conduct research, research with Indigenous populations or their lands, and research on cultural artefacts. The questionnaire can also be requested at the journal’s discretion for any other submissions, even if these conditions are not met. Please find more information on the policy and a link to download a blank copy of the questionnaire here: https://journals.plos.org/plosone/s/best-practices-in-research-reporting. Please upload a completed version of your questionnaire as Supporting Information when you resubmit your manuscript.

This study was developed in Paraguay and the mains, firsts and correspondence authors of this study are Paraguayans. This study does not include the researchers who have travelled to a different country to conduct research, research with Indigenous populations or their lands, and research on cultural artefacts.

4. Please provide additional details regarding participant consent. In the ethics statement in the Methods and online submission information, please ensure that you have specified what type you obtained (for instance, written or verbal, and if verbal, how it was documented and witnessed). If your study included minors, state whether you obtained consent from parents or guardians. If the need for consent was waived by the ethics committee, please include this information.

We added to Ethical considerations section, the consent was written. No women younger than 18 years old were included in the study.

Line 152– 154 “The participants signed a free and informed written consent form before inclusion in the study. No women younger than 18 years old were included in the study”.

5. We note that the grant information you provided in the ‘Funding Information’ and ‘Financial Disclosure’ sections do not match. When you resubmit, please ensure that you provide the correct grant numbers for the awards you received for your study in the ‘Funding Information’ section.

This study was supported by the National Council of Science and Technology (CONACYT) through the PROCIENCIA Program with resources from the Fund for Excellence in Education and Research (FEEI) (RESERCH 15-INV-200), Research, Education and Biotechnologies Applied to Health (IEBAS) Project – MERCOSUR Structural Convergence Fund (FOCEM), COF 03-11, Coordination for the Improvement of Higher Education Personnel (Coordenação de Aperfeiçoamento de Pessoal de Nível Superior - CAPES) - Finance Code 001, Fundação Carlos Chagas Filho de Amparo à Pesquisa do Estado do Rio de Janeiro (FAPERJ), Oswaldo Cruz Institute and Conselho Nacional de Desenvolvimento Científico e Tecnológico (CNPq).

The ‘Funding Information’ and ‘Financial Disclosure’ sections were corrected.

6. Thank you for stating the following financial disclosure:

"Funding information: This work was supported by the National Council of Science and Technology (CONACYT) through the PROCIENCIA Program with resources from the Fund for Excellence in Education and Research (FEEI) (RESERCH 15-INV-200) and by the Research, Education and Biotechnologies Applied to Health (IEBAS) Project – MERCOSUR Structural Convergence Fund (FOCEM), COF 03-11."

"The funders had no role in study design, data collection and analysis, decision to publish, or preparation of the manuscript."

Please include this amended Role of Funder statement in your cover letter; we will change the online submission form on your behalf.

As requested, the information about funder statement was included in the cover letter and we highlight that the funders had no role in study design, data collection and analysis, decision to publish, or preparation of the manuscript.

7. We note that you have indicated that data from this study are available upon request. PLOS only allows data to be available upon request if there are legal or ethical restrictions on sharing data publicly. For more information on unacceptable data access restrictions, please see http://journals.plos.org/plosone/s/data-availability#loc-unacceptable-data-access-restrictions.

In your revised cover letter, please address the following prompts:

a) If there are ethical or legal restrictions on sharing a de-identified data set, please explain them in detail (e.g., data contain potentially sensitive information, data are owned by a third-party organization, etc.) and who has imposed them (e.g., an ethics committee). Please also provide contact information for a data access committee, ethics committee, or other institutional body to which data requests may be sent.

b) If there are no restrictions, please upload the minimal anonymized data set necessary to replicate your study findings as either Supporting Information files or to a stable, public repository and provide us with the relevant URLs, DOIs, or accession numbers. For a list of acceptable repositories, please see http://journals.plos.org/plosone/s/data-availability#loc-recommended-repositories.

We will update your Data Availability statement on your behalf to reflect the information you provide.

There are ethical or legal restrictions on sharing data set, as data contain potentially sensitive information. The contact information of Ethics Committee of Central Laboratory of Public Health of Minister of Public Health and Social Welfare is [biblioteca.lcsp@mspbs.gov.py](mailto:biblioteca.lcsp@mspbs.gov.py) and [cei.lcsp.py@gmail.com](mailto:cei.lcsp.py@gmail.com).

**REVIEWER COMMENTS:**

**Reviewer #1:** Thank you for including me in reviewing this manuscript. The article is that “HIGH PREVALENCE AND CO-INFECTION OF HIGH-RISK HUMAN PAPILLOMAVIRUS GENOTYPES AMONG UNVACCINATED YOUNG WOMEN FROM PARAGUAY”. The sample size is small. The current study does not deliver anything significant new or outbreaking in this matter; at this point, all of this, justifies rejection of the manuscript.

We know that the sample size and the convenience sampling procedure adopted in our study represent important limitations, however these considerations are compensated by the need information of a baseline data on the prevalence and type-specific distribution of HPV infection among unvaccinated young women at the implementation stage of the HPV vaccination program in Paraguay.

1. The introduction is long. Authors should abbreviate some places by combining them. Otherwise, the interest in the subject and the flow of the subject are scattered. For example, sections on vaccines and HPV can be shortened. Places that indicate the purpose of the subject can be highlighted.

We appreciate your suggestions; we cut the introduction and highlighted the most important points.

2. There are minimal grammar errors. I recommend that a native English speaker review the article or use a professional language editing service.

The grammatical errors were checked. The manuscript was revised by a native speak person.

3. ”National primary screening is mainly aimed at women aged 30 years and above; younger women do not frequently attend health services for screening tests.” What is this program like, how is it done?

We changed de sentence.

Lines 80-82 “In Paraguay, primary screening aims to detect precursor lesions of cervical cancer in women over 30 years of age [11], making screening data inadequate for assessing the early impact of the vaccine.”

4. You mentioned the vaccination program that started in 2013. What is this program?

We added more information about the program in the manuscript.

Lines 73–75 “In Paraguay, the quadrivalent HPV vaccine was introduced into the national immunization program in 2013, prioritizing girls ages 9 – 10 years old with 0-1-6-month immunization schedule using school-based vaccination [11].”

5. “which was similar to that reported in other…” Is the age group of all the other studies mentioned in this paragraph the same?

Thank you for your observation, the sentence was changed.

Lines 216 – 220 “In this study, we analyzed a cohort of unvaccinated sexually active women between 18–25 years of age and found an overall HPV prevalence of 54.8%, similar to others cross-sectional studies in South American as Colombia with a prevalence of 60.3% in a same age group [14], and Argentina with a prevalence of 56.3% in 15 – 17 year-old female adolescents [15], showing the high of HPV infections in young women in the region.”

6. As you know, HPV can be eliminated after a certain period of time. There may be elimination, especially under the age of 25. What do the authors think about this and about screening in the early age group?

This information was added in the manuscript.

Line 300-304 “Even though several studies provide evidence that genital HPV infection is very common in young sexually active women and in most cases is a transient and self-limited infection, the evaluation of overall HPV prevalence in this population has been proposed as a short-term endpoint for determining the impact of HPV vaccination in surveillance programs.”

Reviewer #2: The authors conducted a HPV prevalence and genotyping study among unvaccinated females using screening samples in women aged 18-25 years. The data are important because they establish a baseline for monitoring impact of Paraguay’s HPV vaccination program. The study appears to be internally valid and most of the analyses seem to be appropriate. Some edits could improve the readability and further focus the paper on the important information. Although the manuscript is generally well-written, additional attention to English grammar is warranted.

We would like to thank the reviewers for their time and the insightful comments and suggestions, which have improved the manuscript substantially. We believe that the modifications introduced in the revised version of the manuscript address the points raised. The changes incorporated are detailed below and highlighted in the revised report.

Abstract:
First sentence stating “several young women remain unvaccinated” is quite confusing. Isn’t it true that at the time of this study, women in the age group included were never vaccine eligible? Quadrivalent vaccine should be moved from second sentence to first (if virologic surveillance is important, then it would be true for a vaccination program with any formulation).

The sentence was changed.

Lines 17 - 18 “Paraguay launched a human papillomavirus (HPV) vaccination program in 2013, so virological surveillance is important for measuring the effectiveness of HPV vaccines.”

Methods in abstract are almost non-existent – please include some information on the recruitment procedures, including how epidemiologic data and specimen were collected and some basics of the typing assay, such as number of types detected.
The information was added.

Lines 21-28 “This study included 208 women, attending the Central Laboratory of Public Health between May 2020 and December 2021, were invited for testing through social networks and flyers at local health centers and higher education institutes. All participants who agreed to contribute to the study signed a free, prior, and informed consent form and answered a questionnaire that included basic demographic data and determining factors of HPV infection. Human papillomavirus detection and genotyping were conducted using the CLART HPV2 test (Genomica, Madrid, Spain) that allows the individual identification of 35 genotypes.”

Introduction:
The first two sentences of the paper suggest the main reason young women are susceptible to STIs is immaturity of genital tract. While this may be a contributor, the initiation of sexual activity and non-monogamous relationships should not be ignored. Those first two sentences could be deleted – “HPV is the most common STI” is a better start.

As suggested the sentence was changed and the information was deleted.

Lines 42 – 44 “Human papilloma virus (HPV) is the most common sexually transmitted infection (STI) among sexually active young women, being acquired shortly after the first sexual intercourse, with the highest prevalence seen in women aged 25 years or younger [1,2].”

The second paragraph is largely extraneous and most if not all of it could be deleted. Line 53 introduces the term “high-risk types,” which needs to be defined, as it was not introduced in the previous paragraph about the categories of HPV types.

A paragraph was changed, and the information was added in the manuscript.
Lines 45 -49 “Based on epidemiological and biological data HPV 16, 18, 31, 33, 35, 39, 45, 51, 52, 56, 58, 59, 68, 73, and 82 should be considered carcinogenic, or high-risk types (IARC Group 1); HPV26, 53, 66, 67, 68, 70, 73, and 82 probably/possibly carcinogenic or probable/possible high-risk types (IARC Groups 2A and 2B) and HPV 6 and 11 non-carcinogenic, or low-risk types (IARC Group 3)[3].”

The introduction really needs a more complete description of the vaccination program. There is a long paragraph on page 4 beginning with line 64 describing all the internationally approved vaccines, and easily lost at the end of this paragraph is one sentence about Paraguay: “In Paraguay, the quadrivalent Gardasil vaccine was introduced to 10-year-old girls in 2013.” The international information could be shortened, and more information on the Paraguay program added. How many doses of Gardasil do girls receive? In what setting (e.g., schools or primary care)? What has the coverage been? Doing the math, with the study conducted in 2020-21, the females included in the prevalence study had mainly not been vaccine eligible, is this correct? This needs to be spelled out for the reader. On first read, it seemed like the authors had screened out HPV-vaccinated women, which raised questions about vaccination coverage and known herd effects from HPV vaccination. This may still be an issue in the youngest women, and should be addressed in the discussion. It is not a real problem with internal validity, but it needs to be clear what exactly was done and why.

The requested were addressed in the introduction.

Lines 73-75. “ In Paraguay, the quadrivalent HPV vaccine was introduced into the national immunization program in 2013, prioritizing girls ages 9 – 10 years old with 0-1-6-month immunization schedule using school-based vaccination [11].

Terminology with different meanings has been used interchangeably (e.g., on page 5 “impact” in line 90, “efficacy” in line 93, and “effectiveness” in line 95). Impact is the correct term for what this study provides a baseline to assess.

We thank your suggestion. The word efficacy and effectiveness were changed to impact in the text.

Similarly, throughout the paper, the authors refer frequently to vaccines “containing” different HPV types; as there is no virus in the vaccines, it may be more appropriate to say the vaccines “target” different HPV types.

We changed the word containing to target.

Materials and methods:

Study Population:

Please provide more clarity regarding recruitment methods. Do women in this age group normally attend the LCSP for reasons other than study participation? Were women recruited from the LCSP or from other community sites? Was any incentive provided?

We described these points.

Lines 94-101 “. Sexually active women aged 18–25 years, who had not been vaccinated against HPV were invited for screening through social networks and flyers at local health centers and higher education institutes. All invited women who voluntary agreed to participate in this study signed a free, prior, and informed consent form and answered a questionnaire that included basic demographic data and determining factors of HPV infection (age, origin, occupation, educational degree, age at first sexual intercourse, number of sexual partners in the last 12 months, contraceptive methods, sexual habits, alcohol consumption, and tobacco use).”

Were women given results of the HPV testing, and if so, how were they counseled?

We added this information.

Lines 157-160 “The HPV tests results were communicated to the patients by trained health personnel with an extensive health education to inform women and avoid unnecessary additional anxiety and distress, helping to understand HPV and its association with cervical cancer, as well as the importance of screening.

Was the study actually conducted on “randomly selected samples”, as stated on page 5 in line 103, or were a convenience sample of women recruited to participate in this study? These details are important for replicating study methods in a future evaluation of impact.

The recruitment was for convenience, and we emphasized in the study population section.

Lines 92-94 “This cross-sectional study was conducted between May 2020 and December 2021, using convenience sampling strategy, due to the national primary screening is mainly aimed at women aged 30 years and above.”

Statistical analysis:

In the statistical analysis section on page 7 in lines 150-156, please describe the HPV type groupings, and how multiple types were accounted for. For example, if a woman had HPV 18 and HPV 58 detected, would she be included in the numerator of both types? In Figure 1, if a woman had both a HR and a LR type detected, where would she be represented?

We added this explanation in Statistical analysis section.

Lines 141-145 “Participants were counted more than once for assessing the prevalence of HR-HPV, probable/possible HR-HPV, and LR-HPV if they had types from more than one group. Single infection was defined as infection with one HPV type and multiple HPV infections were defined as the detection of two or more HPV types in the same sample.

Figure 1 is potentially misleading because it shows proportion positive for the different type groups out of 100% -- this makes it appear that the prevalence of HR-HPV is higher in older age groups, but that may not be true, and the prevalence is not shown. Consider remaking this figure so that the height of the bars represents total prevalence in each age group.

Thank you for the correction. We changed the Figure 1.

On page 7 in line 151, consider not using “qualitative variables” in the description of the methods. “Participant characteristics” would be a better descriptor.

We changed the sentence.

Lines 145 and 146. “Frequencies and percentages were determined for the participant characteristics.”

Some analyses presented in the results (e.g. odds ratios and 95% CIs) are not described in the stastistical analysis section.

We added the description.

Lines 147-150. “Odd ratios (OR) with 95% confidence interval were calculated to evaluate the association between HPV infection and sociodemographic and behavioral characteristics. For all data analyses and relationships between variables, a value of p<0.05 was considered statistically significant.”

Results:
Table 1 and the corresponding text description are inconsistent. For example, the text refers to percentage who were students without employment, but the table does not include employment status. The text refers to percentage with at least high school education, but the table combines primary and secondary. Table 1 does not show results by age; age is usually one of the strongest predictors of HPV prevalence, and this basic descriptive information needs to be provided.

We agree and describe better the table.

Lines 162-169 “The average age of study participants was 22.5±2.1 years. Of these, 70.2% had university as education level. The mean age of first sexual intercourse was 16.8±2.1 years, and 33.2% reported a previous history of STI. Additionally, 59.1% of the participants reported having fewer than two sexual partners in the last year, 81.7% had no history of smoking, and 72.6% reported alcohol consumption (Table 1). A total of 208 cervical samples were analyzed, all of which yielded a positive result for the human CFTR gene, proving the DNA integrity and good sample quality. HPV DNA was detected in 114 specimens, resulting in an overall prevalence of 54.8% (95% CI 48.0–61.6).”

Consider not reporting odds ratios since this may not be the most appropriate measure of association for this study. Since HPV is not a rare outcome, odds ratios overestimate the strength of the association. Consider not reporting a measure of association at all (relying on comparing percentages with p-values) or consider using prevalence ratios instead. Regardless, the description of this measure of association should be included in the statistical analyses section.

Lines 147- 150. The measure of association was included in the statistical analyses section.

In table 3, please specify N. HPV6 prevalence appears to be off by .1.

N is added. The correction was done.

**Table 3. Frequencies of vaccine-preventable HPV types in young women (N=208).**

| **HPV types** | **N°** | | **% (95% CI)** | |
| --- | --- | --- | --- | --- |
| *2-valent/4-valent/9-valent HPV vaccine types* |  | |  | |
| HPV-16 | 14 | 6.7 (3.3–10.1) | |  |
| HPV-18 | 3 | 1.4 (0.0–3.1) | |  |
| HPV-16/18 | 17 | 8.2 (4.5–11.9) | |  |
| HPV-6 | 8 | 3.8 (1.2–6.5) | |  |
| HPV-11 | 2 | 1.0 (0.0–2.3) | |  |
| HPV-6/11 | 10 | 4.8 (1.9–7.7) | |  |
| HPV-6/11/16/18 | 27 | 13.0 (8.4–17.6) | |  |
| HPV-6/11/16/18/31/33/45/52/58 | 79 | 38.0 (31.4–44.6) | |  |
| *Vaccine cross-protection types* |  |  | |  |
| HPV-31/33/45 | 22 | 10.6 (6.4–14.8) | |  |

In the description of figure 2 on page 11 beginning with line 200, please be clear about how prevalence is determined for single and multiple infections. What is the denominator? Is it 208?

The sentence was completed.

Lines 194 - 195 “A total of 29 different viral types were identified, present in both single and multiple infections among 208 studied women (Fig 2).”

The 208 is the number of women included in this study.

In the legend of figure 2 on page 11 in line 210, HPV 72 LR is not mentioned.
HPV 72 was added to the legend of figures 1 and 2.

Lines 187-192 “Fig 1. Frequency of HR-HPV, pHR-HPV, and LR-HPV in young women from Paraguay (N=208). HR-HPV: High risk HPV types; pHR-HPV: possible high risk; and LR-HPV: low risk HPV types. HR types, including Group 1 IARC, carcinogenic to humans (HPV16, 18, 31, 33, 35, 39, 45, 51, 52, 56, 58, and 59) and Group 2A IARC, probable carcinogenic to humans (HPV68). pHR types, Group 2B IARC, possible carcinogenic to humans (HPV26, 30, 34, 53, 66, 67, 69, 70, 73, 82, 85, and 97). LR types, non-carcinogenic to humans (HPV6, 11, 40, 42, 54, 61, 71, 72, 81, 83, and 84).”

Lines 199-204 “Fig 2. Distribution of viral types among HPV positive genital samples in women with single and multiple infections (N=208). HR-HPV: High risk HPV types; pHR-HPV: possible high risk; and LR-HPV: low risk HPV types. HR types, including Group 1 IARC, carcinogenic to humans (HPV16, 18, 31, 33, 35, 39, 45, 51, 52, 56, 58, and 59) and Group 2A IARC, probable carcinogenic to humans (HPV68). pHR types, Group 2B IARC, possible carcinogenic to humans (HPV26, 30, 34, 53, 66, 67, 69, 70, 73, 82, 85, and 97). LR types, non-carcinogenic to humans (HPV6, 11, 40, 42, 54, 61, 71, 72, 81, 83, and 84).”

Discussion:
Generally, the discussion could be trimmed to focus on the most salient information. For example, the paragraph on multiple types that starts on page 13 with line 245 may be extraneous.

This sentence was eliminated.

The last paragraph before the conclusion beginning on page 15 with line 295 is confusing, with discussion expanding to other STIs. This paragraph may not be needed as well.

This sentence was re-written.

Lines 245-246 “Other STI like Chlamydia trachomatis have been associated with cell inflammatory processes in the cervix, facilitating HPV entry and reducing its elimination [38–39].”

A very high number of references is cited (76), and the authors should focus on those that are most important to the topic of establishing a baseline HPV prevalence in Paraguay.

Some references were eliminated.

On page 14 beginning with line 271, consider adding more context to the discussion of HPV 58. While HPV 58 has a high prevalence in Paraguay and in other countries worldwide, before suggesting it be given equal consideration to HPV-16, its degree of carcinogenicity should be considered. Most HPV-attributable cancers are caused by HPV-16 and 18. International studies found HPV-58 in 2.2% (de Sanjose JNCI Cancer Spectr 2018) and 4.4% (Guan et al., IJC 2012) of invasive cervical cancers, far below the contributions of the high-risk types targeted by the quadrivalent vaccine.
We changed the paragraph.

Lines 267 – 274 “It is established that persistent infections by HPV-16 have a higher probability of causing precancerous lesions than infection with other viral types [49, 50]. Women with normal cytology and persistent HPV-16 infection have an approximately 30% chance of developing high-grade lesions in a 12-month period [51]. HPV-58 is also included in high-risk group and has been frequently reported in cervical cancer and high-grade lesions in several regions of the world [52], such as Asia [53, 54], Mexico [56, 56], Costa Rica [44], and Brazil [57]. Given the wide circulation of HPV-58 in the population studied and the evidence of its oncogenic potential, particular attention should be given to HPV-58 infection.”

On page 15 in line 285, it is confusing to state that HPV 58 is not vaccine preventable (it is targeted by Gardasil-9); better to say it is not targeted by the vaccine currently in use in Paraguay’s vaccination program.

The suggestion was taken into account.

Lines 284-285 “HPV-58, not targeted by the vaccine currently in use in Paraguay’s vaccination program was the most prevalent type in this studied group.

The discussion does not appear to include a paragraph on limitations of the study. It is important to at least acknowledge limitations of the study population not being representative of the population of Paraguay or Asuncion and how recruitment of a comparable population for a follow-up study to measure vaccine impact would be accomplished.

The limitations were added.

Lines 294 – 304 “The present study had some limitations as the small sample size of population studied, for this reason the results cannot be extrapolated to the total young women from Paraguay. The recruitment strategy by social networks and flyers at local health centers and higher education institutes may have induced a selection bias. Although this was not a population-based study, the results are particularly important, they results report here in provide information regarding HPV prevalence in groups that are not often included in epidemiological surveys or cervical cancer prevention programs.”

On page 16, in the conclusion in lines 308-309, consider deleting “Considering the high prevalence, multiple infections, and non-vaccine-type HPV infections“ from the second sentence. The data provide a baseline regardless of these considerations.

The suggestion was taken into account.

Lines 305 - 309. “In conclusion, this is the first report on the prevalence of HPV and its genotype distribution, including HPV types targeted by current vaccines, in unvaccinated young sexually active women from Paraguay. These data provide the baseline against which to compare future HPV prevalence of vaccine and non-vaccine-types, allowing the estimation of the impact of the national HPV immunization program.”

Reviewer #3: 1) The title the authors picked is an interesting and pioneer base line focus of interest specific to Paraguay. Authors used simple random sampling to select 208 women aged 18-25. Do you have an explanation or a local reference for the statement on line 106 "younger women..."?

We apologize because we used a convenience sampling strategy, that is noted in Line 93. Then the paragraph was changed, and the local reference was added.

Lines 92 – 94 “This cross-sectional study was conducted between May 2020 and December 2021, using convenience sampling strategy, due to the national primary screening is mainly aimed at women aged 30 years and above [11].”

Secondly, what is the conclusion you have drawn from this research? Furthermore, what are your recommendations?

Lines 305-306 “In conclusion, this is the first report on the prevalence of HPV and its genotype distribution, including HPV types targeted by current vaccines, in unvaccinated young sexually active women from Paraguay. These data provide the baseline against which to compare future HPV prevalence of vaccine and non-vaccine-types, allowing the estimation of the impact of the national HPV immunization program.”

2) Although looking grossly sound, I have one questions regarding the result/analysis section. Why is the summation of those with History of STI and Negative test not 94; it is 91 in your case?

We apologized. It was a typing mistake.

Overall, it is a very well written manuscript discussing a hot issue mainly concerning developing countries like Paraguay. It is a good initiative but the conclusion and recommendation needed further elaboration than short statements.

Thank for your suggestions. The conclusion was improved.

Reviewer #4: This study aimed to estimate the type-specific HPV frequency in unvaccinated women aged 18–25 years in the metropolitan area of Asuncion as a baseline for monitoring the HPV vaccination program. I have reviewed the manuscript and would like to suggest the following changes.

Discussion :

The first paragraph should mention the noteworthy findings of the study. The authors should compare and contrast these findings with other studies in subsequent paragraphs. Also, author should add a paragraph regarding limitations and future implication of this study. The conclusion should be a separate paragraph. The references are very numerous, it may be necessary to reduce a little.

All suggestion were done along the discussion and the numberer of references was reduced.

Reviewer #5: The paper was on the HIGH PREVALENCE AND CO-INFECTION OF HIGH-RISK HUMAN PAPILLOMAVIRUS GENOTYPES AMONG UNVACCINATED YOUNG WOMEN FROM PARAGUAY. The results showed important information for public health.

However, as the title was on prevalence, there were no mentioned on the estimation of sample size as N=208 was small. the demographic data showed that the participants were actually moderate risk eg. only 24.0 % used condom, 33% reported STI, 40.9% had reported 2 or more partners. These should add in the discussion, all these are risk factors for acquiring HPV. That's why the results reported quite high high risk HPV types.

All suggestion were performed along the text.

Line 294 -295 The present study had some limitations as the small sample size of population studied, for this reason the results cannot be extrapolated to the total young women from Paraguay.

Lines 299- 239 – “Although the demographic data and all risk factors for acquiring HPV showed that the participants were moderate risk e.g., only 24.0 % used condom, 33% reported STI, 40.9% had reported 2 or more partners, the results reported high risk HPV types. At least one type of HR-HPV was found in 42.3% of young women, with HR-HPV prevalence ranging from 18.7%–45.7% among women under 25 years old worldwide [24-31]. A similar rate (42.2%) has been observed in regional surveys conducted in Colombia and Argentina [14,15]. However, an elevated HR-HPV infection rate in young women does not necessarily lead to the development of precancerous lesions and cancer. The decreasing frequency of infection with age has been demonstrated in national, regional, and global studies, indicating the self-limited nature and control of the infection by the action of the host's immune system in most cases [32,33, 21-23].”

***********************************************************************
