# Supplementary material for: High prevalence and co-infection of high-risk Human Papillomavirus genotypes among unvaccinated young women from Paraguay
Source: PLoS One. 2023 Apr 6;18(4):e0283542. doi: 10.1371/journal.pone.0283542 (PMC10079089; doi:10.1371/journal.pone.0283542)
Supplement: Attachment — Submitted filename: Response to Reviewers 28th Feb PONE-D-22-28769R1.docx [file pone.0283542.s002.docx]

February 28^th^, 2023

**Manuscript number:** PONE-D-22-28769R1

Dear Academic Editor Andrea Giannini

Thank you for giving us the opportunity to submit a revised draft of the manuscript "High prevalence and co-infection of high-risk *Human Papillomavirus* genotypes among unvaccinated young women from Paraguay”. We appreciate the time and effort that you and the reviewers dedicated to providing feedback on our manuscript and are grateful for the insightful comments on and valuable improvements to our paper.

We have incorporated most of the suggestions made by the reviewers. Those changes are highlighted within the manuscript. Please see below, in blue, for a point-by-point response to the reviewers’ comments and concerns. All page numbers refer to the revised manuscript file with tracked changes.

We look forward to your response and hope you and the reviewers will be satisfied with the revised manuscript and will find it suitable for publication.

Sincerely yours,

María Liz Bobadilla

Lab. of Immunology, Central Laboratory of Public Health/Minister of Public Health and Social Welfare, Asunción, Paraguay.

Lab. De Virologia e Parasitologia Molecular IOC/FIOCRUZ, Rio de Janeiro, Brazil.

**REVIEW COMMENTS TO THE AUTHOR**

**Reviewer #1:** The authors made several corrections. But as I said in my first review: The current study does not deliver anything significant new or outbreaking in this matter.

We respect your opinion, but as you know the incidence of cervical cancer in Paraguay is higher than other countries in the region and an important component of its prevention is the HPV vaccination. So, Paraguay, like many other countries worldwide, has incorporated the HPV vaccine into its national immunization program, requiring a virological surveillance to monitor changes in the virus transmission. We know that the population-based cohort studies are the most appropriate to assess the impact of HPV vaccination; however, the feasibility of conducting these studies is limited in low resources countries, thus cross-sectional surveys in sentinel populations could be a suitable alternative.

For hence this study even its important limitations, such as the small sample size and the convenience recruitment strategy, the results are particularly important, providing information regarding HPV prevalence in groups that are no often included in national cervical cancer prevention programs. These data provide the baseline against which to compare future HPV prevalence of vaccine and non-vaccine-types, allowing the short-term estimation of the impact of the national HPV immunization program.

These explanations were added in the discussion.

**Lines 342-351.** We know that the population-based cohort studies are the most appropriate to assess the impact of HPV vaccination; however, the feasibility of conducting these studies is limited in low resources countries, thus cross-sectional surveys in sentinel populations could be a suitable alternative.

For hence this study even its important limitations, such as the small sample size and the convenience recruitment strategy, the results are particularly important, providing information regarding HPV prevalence in groups that are no often included in national cervical cancer prevention programs. These data provide the baseline against which to compare future HPV prevalence of vaccine and non-vaccine-types, allowing the short-term estimation of the impact of the national HPV immunization program.

**Reviewer #4:** (No Response)

**Reviewer #6:** I read with great interest the manuscript, which falls within the aim of this Journal. In my honest opinion, the topic is interesting enough to attract the readers’ attention. Nevertheless, the authors should clarify some points and improve the discussion, as suggested below. Authors should consider the following recommendations:

- Manuscript should be further revised in order to correct some typos and improve style.

Thank you for your observation. The text was revised.

- To date, several lines of evidence support the possibility to use specific biomarkers to identify early-stage cervical cancer and, in this way, offer a better prognosis to the patients. This point deserves to be discussed, referring to: PMID: 28918603; PMID: 35549629.

- I would recommend to stress novel pieces of evidence about high-risk HPV-negative high-grade cervical dysplasia, which seems to have more favorable outcomes than patients with documented high-risk-HPV infection (PMID: 35742340; PMID: 33514481).

We appreciate your suggestions, we read the mentioned references and we added some important points in the introduction:

**Lines 63 – 69.** Cervical cancer is a disease which reflects inequities among different populations depending on the availability of a national vaccination program and population-based cervical cancer screening, and access to quality treatment. In 2020, the World Health Organization (WHO) called for action to eliminate cervical cancer as a public health problem. With three key strategies and clear 2030 targets: 90% of girls fully vaccinated with HPV vaccine by 15 years of age, 70% of women screened using a high-performance test by 35 years of age and again by 45 years, and 90% of women identified with cervical disease treated [8].

**Line 70.** The primary prevention approach focuses on preventing disease before it develops.

**Lines 79 – 92.** Secondary prevention, based on cervical screening program, is widely implemented to prevent. The adoption of Pap smear and HPV testing resulted in a significant increase in the diagnosis of cervical dysplasia and a significant decrease in cervical cancer, mainly in more developed countries. New screening strategies, such as HPV self-sampling, and the use of artificial intelligence in colposcopy assessment, must be disseminated in a near future [11].

Some markers, including p16 ink4a, p16, E-cadherin, Ki67, pRb, and p53, have been found to be useful in identifying intra-epithelial lesions that are more likely to develop into invasive lesions. Others, such as CEA, SCC-Ag, CD44, were developed to detect invasive forms. These biomarkers represent a promising diagnostic, prognostic and dynamic tool and can also be easily performed in the management of cervical cancer [12].

And tertiary prevention in cervical cancer includes its treatment with surgery (radical hysterectomy), radiotherapy, and systemic treatments (chemotherapy, bevacizumab, and immunotherapy) [11]. Minimally invasive surgery has been studied and associated with advantages in the treatment of various gynecological cancers [13].

**Reviewer #7:** I read with great interest the Manuscript titled "HIGH PREVALENCE AND CO-INFECTION OF HIGH-RISK HUMAN PAPILLOMAVIRUS GENOTYPES AMONG UNVACCINATED YOUNG WOMEN FROM PARAGUAY " which falls within the aim of the Journal.
In my honest opinion, the topic is interesting enough to attract the readers’ attention. Methodology is accurate and conclusions are supported by the data analysis. Nevertheless, authors should clarify some point and improve the discussion citing relevant and novel key articles about the topic.

-The introduction should be extended and completed. I find interesting a reference to the efforts made for the prevention and early diagnosis of gynecological cancers (see PMID: 36141217).

Based on the suggested reference, we have added information on new screening strategies and certain biological markers that may be useful in early diagnosis and prognosis of cervical dysplasia, and new approaches to the treatment pre-cancerous lesions and cervical cancer.

**Lines 79 – 92.** Secondary prevention, based on cervical screening program, is widely implemented. The adoption of Pap smear and HPV testing resulted in a significant increase in the diagnosis of cervical dysplasia and a significant decrease in cervical cancer, mainly in more developed countries. New screening strategies, such as HPV self-sampling, and the use of artificial intelligence in colposcopy assessment, must be disseminated in a near future [11].

Some markers, including p16 ink4a, p16, E-cadherin, Ki67, pRb, and p53, have been found to be useful in identifying intra-epithelial lesions that are more likely to develop into invasive lesions. Others, such as CEA, SCC-Ag, CD44, were developed to detect invasive forms. These biomarkers represent a promising diagnostic, prognostic and dynamic tool and can also be easily performed in the management of cervical cancer [12].

And tertiary prevention in cervical cancer includes its treatment with surgery (radical hysterectomy), radiotherapy, and systemic treatments (chemotherapy, bevacizumab, and immunotherapy) [11]. Minimally invasive surgery has been studied and associated with advantages in the treatment of various gynecological cancers [13].

- Although it is a retrospective analysis, inclusion/exclusion criteria should be better clarified by extending their description.

We explained more clearly the inclusion/exclusion criteria.

**Lines 114 – 117.** Sexually active women aged 18–25 years, who had not been vaccinated against HPV were invited for screening through social networks and flyers at local health centers and higher education institutes. Exclusion criteria were pregnancy, nonsexual onset at the time of the study and have been received HPV vaccine.

- Discussions can be expanded and improved by citing relevant articles (I suggest authors to read and insert in references the following article PMID: 35742340).
Considered all this points, I think it could be of interest for the readers and, in my opinion, it deserves the priority to be published after minor revisions.

Discussions have been expanded and improved as suggested, with the follow changes:

**Lines 235 – 253.** The WHO global strategy for the elimination of cervical cancer as a public health problem proposes a multidisciplinary approach that includes community education, social mobilization, vaccination, detection, treatment, and palliative care.

HPV vaccination in combination with timely detection and appropriate treatment of cervical cancer precursor lesions is considered to have the potential to achieve the elimination of this disease, defined by the WHO as fewer than 4 new cases per 100,000 women-years [8].

With the introduction of the HPV vaccine, changes in the transmission dynamics of the virus are expected, making necessary to monitor changes in the prevalence of HPV and the patterns of HPV-associated diseases. Population-based cohort studies are the most appropriate to assess the impact of HPV vaccination; however, the feasibility of conducting these studies is limited in low- and middle-income countries, so cross-sectional surveys in sentinel populations could be a suitable alternative [8].

Paraguay, like many other countries worldwide, has incorporated the HPV vaccine into its national immunization program, requiring a virological surveillance to measure its impact. It can provide an early measure of vaccine effectiveness by quantifying the magnitude of reduction in HPV type-specific prevalence, including possible evidence of herd and cross protection and provide timely assessment of vaccination strategy. The principle of HPV prevalence surveillance is to recruit samples in age groups at risk of HPV exposure and conduct repeat-cross sectional analyses [16].

**Lines 326 – 329.** Have been reported women immunized with quadrivalent vaccine at young age who still developed cervical intraepithelial neoplasia with evidence that these lesions are commonly associated with HPV types not targeted by this vaccine, but some of the HPV types associated with these lesions in vaccinated women are targeted by the Gardasil-9 [59].

**Lines 332 – 335.** Therefore, the high prevalence of HPV types targeted by nonavalent HPV vaccines could support its introduction in the national immunization program to achieve greater coverage, considering that the preventable proportion of cervical cancers is estimated to increase to 90% [60].

**Lines 342 – 351.** We know that the population-based cohort studies are the most appropriate to assess the impact of HPV vaccination; however, the feasibility of conducting these studies is limited in low resources countries, thus cross-sectional surveys in sentinel populations could be a suitable alternative.

For hence this study even its important limitations, such as the small sample size and the convenience recruitment strategy, the results are particularly important, providing information regarding HPV prevalence in groups that are no often included in national cervical cancer prevention programs. These data provide the baseline against which to compare future HPV prevalence of vaccine and non-vaccine-types, allowing the short-term estimation of the impact of the national HPV immunization program.

**Lines 356 – 365.** This research could provide key information to healthcare policy makers about the importance of increasing HPV vaccination coverage in national immunization programs. The results should be combined with other studies in cohorts of vaccinated women to investigate the reduction of types 16 and 18, as well as their possible replacement by other genotypes, thus evaluating the impact of the implementation of HPV vaccination in Paraguay and compare the results with other countries.

In addition, these data can improve the acceptance of the HPV vaccine in the Paraguayan population, highlighting the high prevalence of HPV, especially the high-risk types, observed in unvaccinated young women. Increased public awareness of the benefits of HPV vaccination for cervical cancer prevention could increase public support for immunization.

**Lines 370 – 373.** Surveillance and monitoring of changes in viral transmission that lead to modification of HPV type-specific prevalence and the patters of HPV-associated disease are crucial to progress toward the global elimination of cervical cancer.

***********************************************************************
